# ﻿Discovery of *Synuchuscongruus* (Morawitz, 1862) (Coleoptera, Carabidae, Sphodrini) in Europe

**DOI:** 10.3897/zookeys.1247.151146

**Published:** 2025-07-24

**Authors:** Mikalai Kazulka, Alexander Anichtchenko, Oleg Aleksandrowicz

**Affiliations:** 1 Scientific and Practical Center of the National Academy of Sciences of Belarus for Biological Resources, Akademicheskaya 27, 220072 Minsk, Belarus Scientific and Practical Center of the National Academy of Sciences of Belarus for Biological Resources Minsk Belarus; 2 Institute of Systematic Biology, Daugavpils University, Vienibas iela 13-229, LV-5400 Daugavpils, Latvia Daugavpils University Daugavpils Latvia; 3 Institute of Biology, Pomeranian University in Słupsk, Arciszewski 22, 76-200, Słupsk, Poland Pomeranian University in Słupsk Słupsk Poland

**Keywords:** Carabid beetle, Europe, identification key, new records

## Abstract

The eastern Palearctic carabid beetle *Synuchuscongruus* (Morawitz, 1862) is recorded from Belarus, Latvia, and Poland. Images of habitus, morphological features, and aedeagi, as well as an updated key to the *Synuchus* species of Europe, are provided.

## ﻿Introduction

The genus *Synuchus* Gyllenhal, 1810, a member of the family Carabidae, includes 86 species found in the Palearctic, Oriental, and Nearctic regions (Bousquet 2012; Hovorka 2017; Kurian and Thomas 2022). The species diversity in the genus is unevenly distributed across its range, with the vast majority of species known from East Asia, particularly in Japan, Russian Far East, China, and the Korean Peninsula. Until now, only one species—*Synuchusvivalis* (Illiger, 1798)—was known to inhabit Europe. It occurs from the Iberian Peninsula, France and the British Isles in the west, Israel and Turkey in the south, through Central and Northern Europe, European Russia, the Caucasus, Siberia, and Central Asia to the Russian Far East, north-east China, and Japan (Hovorka 2017), where it can be found mainly in open habitats (Koch 1989; Lindroth 1992; Magura et al. 2000; Aleksandrowicz 2014) from lowlands to rather high elevations in mountains.

The present paper provides data on the surprising presence of *Synuchuscongruus* (Morawitz, 1862) in Central and Eastern Europe. Considering its probable widespread distribution in the region, we provide diagnostic characters and an updated key for the identification of *Synuchus* species of Europe.

## ﻿Materials and methods

The study is based on specimens collected during various field trips in Belarus, Poland, and Latvia over the last several years. In addition, all available *Synuchus* specimens preserved in a number of institutional and private collections in the listed countries were examined for the presence of *S.congruus*. The collections referred to in this study are abbreviated as follows:

**SPCM**Scientific and Practical Center of the National Academy of Sciences of Belarus for Biological Resources, Minsk, Belarus

**DUBC** Daugavpils University, Institute of Life Sciences and Technology, Daugavpils, Latvia

**PUIB** Pomeranian University in Słupsk, Institute of Biology, Słupsk, Poland

**FRI** Forest Research Institute, Białowieża, Poland

**cKuz** collection of V. Kuznetzov, Minsk, Belarus

An updated key to European *Synuchus* species was prepared on the basis of a morphological study of collected beetles and existing keys (Lindroth 1956; Lafer 1989). The distributional map was made using SimpleMappr (Shorthouse 2010). Photographs of the beetles and their aedeagi were made using a Canon EoS 6D digital camera with a Canon MP-E 65 mm macro lens, StackShot system and helicon Focus auto montage. Final post-processing of images was done in Adobe Photoshop.

## ﻿Results

### ﻿ Synuchus congruus

Taxon classificationAnimaliaColeopteraCarabidae

(Morawitz, 1862)

DD2814B4-A91E-5A48-BFA1-AEC2C826452C

Taphria congrua Morawitz, 1862
Synuchus latus Tschitschérine, 1893

#### Taxonomic remarks.

**Figures 1, 2.**
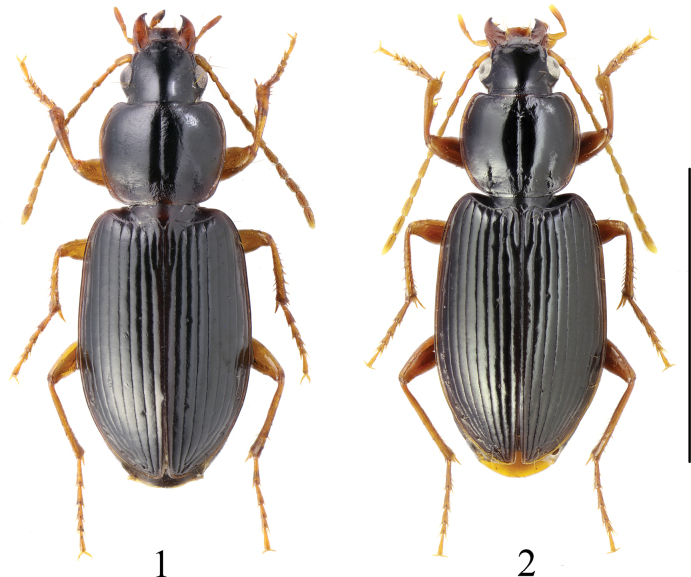
Habitus of *Synuchus*. **1.***S.vivalis*; **2.***S.congruus*. Scale bar: 5 mm.

**Figures 3–6.**
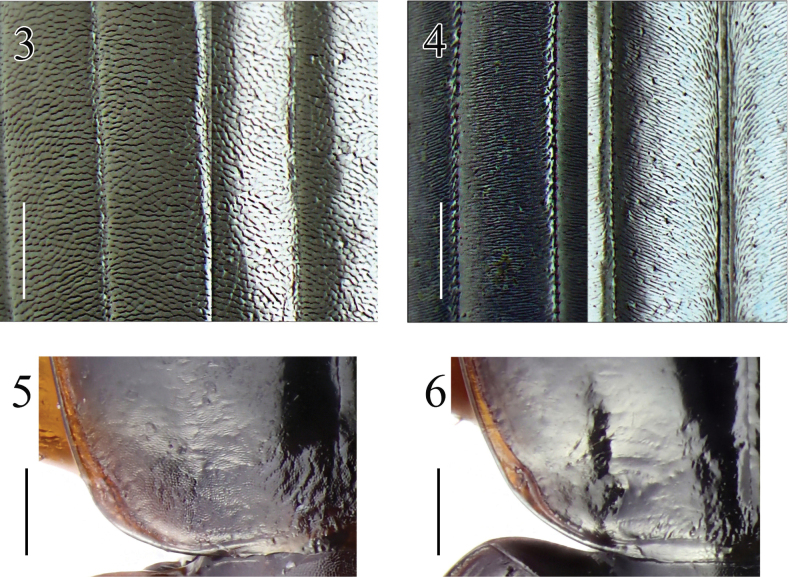
Microreticulation in *Synuchus* (males). **3, 4.** on elytra; **5, 6.** on base of pronotum; **3, 5.***S.vivalis*; **4, 6.***S.congruus*. Scale bars: 0.15 mm (**3, 4**); 0.3 mm (**5, 6**).

**Figures 7, 8.**
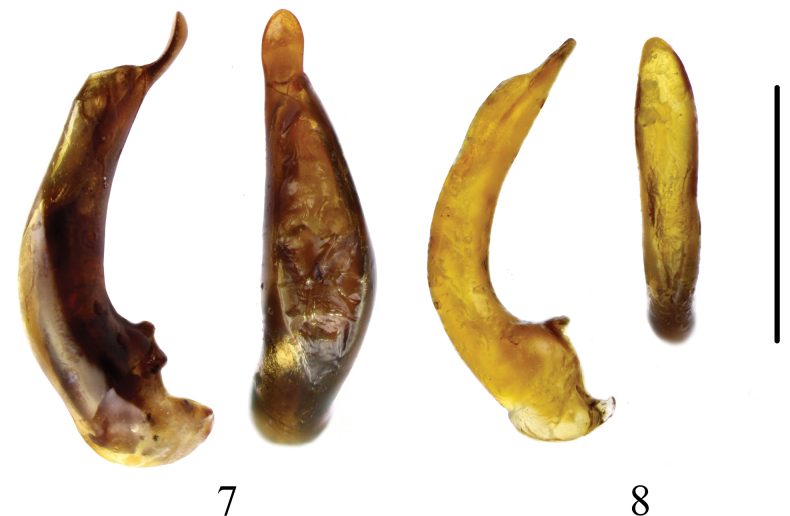
Aedeagus of *Synuchus* in lateral and dorsal view. **7.***S.vivalis*; **8.***S.congruus*. Scale bar: 1 mm.

The genus *Synuchus* has been represented in Europe by a single widespread species *Synuchusvivalis*. It can be easily distinguished from other European Sphodrini Laporte, 1834 by the following features: dilated last labial palpomere, serrated tarsal claws, rounded base of the pronotum, prosternal process without a border, and characteristic shape of the aedeagus (Lindroth 1956; Aßmann 2004). Despite the extreme similarity in habitus (Figs 1, 2), the morphology of numerous specimens recorded over the past few years differed from that characteristic for *S.vivalis*. They have transverse reticulation on the elytra (rounded/isodiametric in *S.vivalis*) (Figs 3, 4), rather faint reticulation on the base and disc of pronotum (clearly visible in *S.vivalis*) (Figs 5, 6), weaker reticulation on forehead and vertex (clearly visible in *S.vivalis*), and deeper lateral furrows on middle and hind tarsi (weaker in *S.vivalis*). The differences also concern the shape of the aedeagus, the apex of which is short and rounded, and the median lobe in dorsal view is almost symmetrical (in *S.vivalis* the apex is elongated and curved upward, and the median lobe in dorsal view is asymmetrical and enlarged to the right) (Figs 7, 8). Careful examination of these specimens showed that their morphology corresponds to the characteristics of *S.congruus* given by Lindroth (1956) and Lafer (1989), and they are clearly conspecific with specimens of *S.congruus* from Eastern Siberia and the Russian Far East.

#### Collection and habitat data.

**Figure 9.**
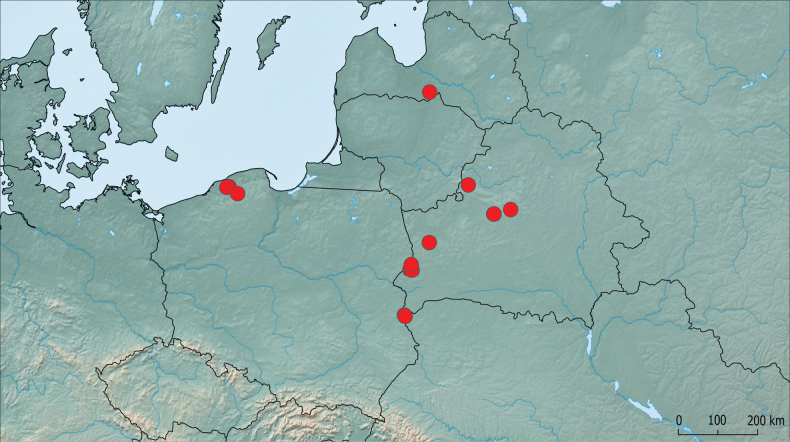
Occurrence map of *Synuchuscongruus* in Belarus, Latvia, and Poland.

Specimens of *S.congruus* were collected from multiple locations in western and central Belarus, northern and eastern Poland, and Latvia (Fig. 9). They were mainly found in forests, with several specimens in fallow fields and meadows. One was caught in bush thickets near a river. Almost all specimens were collected using pitfall traps or sifted from leaf litter, except for one, which was found in the fruiting body of *Pleurotus* sp. on a fallow oak tree in an old-growth oak forest.

#### Locality data.

Belarus – **Minsk region**: Dzerzhinsky distr., near Volmechka vlg. (UTM code: MV96), 53°50'44"N, 26°55'15"E, meadow, pitfall traps: 1 ex., 9.X.2011, leg. V. Lukin (SPCM) • Dzerzhinsky distr., near Volmechka vlg. (MV96), 53°50'47"N, 26°56'16"E, meadow, pitfall traps: 1 ex., 9.X.2011, leg. V. Lukin (SPCM) • Minsk (NV37), 53°56'35"N, 27°33'11"E, municipal woodland, hand collecting: 1 ex., 1.VIII.2020, leg. V. Kuznetzov (cKuz) – **Brest region**: Kamenets dist., near Lyatskie vlg. (FD93), Białowieża Forest (Belovezhskaya Pushcha National Park), 52°36'56"N, 23°52'30"E, birch forest, pitfall traps: 2 ex., 8.IX–11.X.2017, leg. O. Prischepchik (SPCM) • Kamenets dist., near Lyatskie vlg. (FD93), Białowieża Forest, 52°35'19"N, 23°51'42"E, hornbeam forest, pitfall traps: 1 ex., 26.X–7.XI.2024, leg. M. Kazulka (SPCM) • Kamenets dist., near Lyatskie vlg. (FD93), Białowieża Forest, 52°36'11"N, 23°51'39"E, oak forest, pitfall traps: 1 ex., 26.X–7.XI.2024, leg. M. Kazulka (SPCM) • Kamenets dist., near Lyatskie vlg. (FD93), Białowieża Forest, 52°35'14"N, 23°52'33"E, oak-hornbeam forest, pitfall traps: 1 ex., 26.X–7.XI.2024, leg. M. Kazulka (SPCM) • Kamenets dist., near Kamenyuki vlg. (FD83), Białowieża Forest, 52°36'18"N, 23°47'48"E, oak forest, hand collecting (on *Pleurotus* sp.): 1 ex., 18.VIII.2021; pitfall traps: 4 ex., 26.X–7.XI.2024, leg. M. Kazulka (SPCM) • Brest dist., Tomashovka vlg. (FC71), Pribuzhskoe Polesie Biosphere Reserve, 51°33'18"N, 23°35'30"E, pine forest, pitfall traps: 1 ex., 16.III–17.IV.2022, leg. V. Kuznetzov (cKuz) • Brest dist., near Orkhovo vlg. (FC81), Pribuzhskoe Polesie Biosphere Reserve, 51°31'29"N, 23°37'17"E, broadleaved forest, pitfall traps: 13 ex., 16–23.X.2021; 10 ex., 23–30.X.2021; 31 ex., 31.X–16.XI.2021; 11 ex., 16.XI.2021–12.II.2022, leg. V. Kuznetzov (cKuz) • Brest dist., near Orkhovo vlg. (FC81), Pribuzhskoe Polesie Biosphere Reserve, 51°31'55"N, 23°36'39"E, broadleaved forest, hand collecting: 1 ex., 16.X.2021; pitfall traps: 1 ex., 27.III–11.IV.2021, leg. V. Kuznetzov (cKuz); 7 ex., 30.X–6.XI.2023, leg. M. Kazulka (SPCM) – **Grodno region**: Volkovysk dist., near Zvezdnaya vlg. (LU39), Zamkavy Les Landscape Reserve, 53°12'49"N, 24°31'09"E, oak forest, pitfall traps: 1 ex., 24.VI–4.VIII.2022; 16 ex., 4.VIII–6.IX.2022; 11 ex., 6.IX–6.X.2022, leg. M. Kazulka (SPCM) • Oshmyany dist., near Pratskovshchina vlg. (MA33), 54°28'44"N, 25°58'37"E, oak-aspen forest, pitfall traps: 4 ex., 5.IX–5.X.2022, leg. M. Kazulka (SPCM).

Latvia – Bārbele parish, Bauska municipality (LC45), 56°27'00"N, 24°32'04"E, bush thickets near a river, pitfall trap: 1 ex., 8–11.VIII.2024 (DUBC).

Poland – **Pomeranian Voivodeship**: near Widzino vlg. (XA23), 54°25'41"N, 16°58'23"E, fallow field, pitfall traps: 1 ex., 22.V–5.VI.2007; 1 ex., 22.VII–9.VIII.2007; 5 ex., 20.IX–8.X.2007; 4 ex., 8.X–3.XI.2007; 6 ex., 3–20.XI.2007, leg. M. Czyżykowska (PUIB) • Słupsk city (XA33), 54°26'38"N, 17°03'23"E, oak-beech municipal forest, pitfall trap: 1 ex., 15–30.XI.2009, leg. O. Aleksandrowicz (PUIB) • Słupsk city (XA23), 54°29'01"N, 16°59'30"E, fallow field, pitfall trap: 1 ex., 1–20.XI.2012, leg I. Mrozowicz (PUIB) • Near Niepoględzie vlg. (XA51), 54°17'27"N, 17°22'38"E, wooded strip (hornbeam forest), pitfall traps: 9 ex., 15.X–11.XI.2010, leg. M. Janusiak (PUIB) – **Podlaskie Voivodeship**: near Białowieża vlg. (FD94), Białowieża Forest (Białowieża National Park), comp. 399C, 52°43'04"N, 23°50'52"E, oak–hornbeam forest, pitfall traps: 2 ex., 31.VII.2012, leg. J. Gutowski (FRI).

## ﻿Discussion

*Synuchuscongruus* was originally described as *Taphriacongrua* by Morawitz (1862) from the “Bureja-Gebirge” (Bureinsky Ridge near Amur River, Russian Far East). In 1893, Tschitschérine described *Synuchuslatus* from the Zabaykalsky Krai, Russia. Habu (1955) recognized that the two forms were conspecific and proposed their synonymy, which was consistent with the views of Lindroth (1956). The previously known range of the species includes Russia (southern Kuril Islands, southern Sakhalin, Primorskii Krai, southern Khabarovskii Krai, Jewish Autonomous Region, Amurskaya Oblast’, Transbaikalia, southern Siberia, southern Ural), Kazakhstan, North and South Korea, Japan, and the northwestern, northern, and northeastern regions of China (Šustek 2003; Sundukov and Makarov 2013; Saimova et al. 2020; Zhu 2022).

New data show that *S.congruus* has spread across Eastern and Central Europe. It has been recorded from Słupsk in the west to Minsk in the east, from Latvia in the north to Brest Polesie in the south-western Belarus. Although ground beetles have been one of the most active areas in beetle research in Europe over the past decades, what is most surprising is that it was overlooked in the region for many years and probably confused with *S.vivalis*, since the two species are extremely similar in habitus. In fact, a careful study of the carabids stored in the collections of the SPCM and PUIB showed that several specimens belonging to this species were initially misidentified as *S.vivalis*.

The earliest detected specimen of *S.congruus* from the studied collections was sampled in northern Poland in 2007. This suggests that the species was introduced into Europe prior to that year, although the exact time and point of entry are unlikely to be accurately determined. The study of beetles preserved in other collections, primarily those sampled from Poland, Germany, the Baltic countries, and the northwestern part of European Russia, could provide valuable insights. The method of introduction is also unknown, but may be due to the importation of plant stock or associated materials, as is known for other alien ground beetle species, such as *Pterostichusvagus* in Czech Republic (Hůrka and Šustek 1995) (previously recognized as *P.caspius* until its revision by Muilwijk et al. 2024), *Leistusfulvibarbis* in Norway (Westergaard et al. 2015), and multiple cases from North America (Kavanaugh and Erwin 1985; Spence and Spence 1988; Kavanaugh and LaBonte 2008). The ability for active flight allowed *S.congruus* to spread further in Europe.

In its native range, *S.congruus* is found in coniferous and deciduous forests, as well as on forest edges, clearings, bush thickets, and meadows, along riverbanks, and in wetlands (Lafer 1976; Sundukov 2009; Rogatnykh 2009; Shabalin et al. 2010; Sundukov and Makarov 2013; Sundukov 2024). In our study, the species was collected both in forest and open habitats of varying degrees of naturalness: from old-growth oak and hornbeam forests in Białowieża Forest and semi-natural oak-dominated forests to municipal woodlands, fallow fields and wooded strips surrounded by agricultural lands. It was observed from spring to late autumn. In several localities, the species was collected together with *S.vivalis*.

Future studies will help to accumulate data on the distribution of *S.congruus* in Europe, especially in neighboring countries (Germany, Ukraine, Lithuania, Estonia, and the European part of Russia) and to clarify its habitat and ecological preferences.

### ﻿Identification key to the species of genus *Synuchus* in Europe

**Table:** 

1	Elytral surface and base of pronotum with isodiametric microsculpture (Fig. 3). Base of pronotum with clearly visible isodiametric meshes (Fig. 5). Meso- and metatarsomeres 1–3 with shallow furrows. Apex of aedeagus elongated and dilated, in lateral view slightly bends dorsally. Median lobe in dorsal view asymmetrical and enlarged to right (Fig. 7). Brachypterous or macropterous. Brownish to piceous-black, side-margins of pronotum and elytra usually brighter. Body length 5.8–9 mm	***S.vivalis*** (Illiger, 1798)
–	Elytral surface with transverse microsculpture (Fig. 4). Base of pronotum with rather faint microreticulation thus is shinier (Fig. 6). Meso- and metatarsomeres 1–3 with deep furrows. Apex of aedeagus shorter and rounded. Median lobe in dorsal view more or less symmetrical (Fig. 8). Macropterous. Piceous-black, side-margins of pronotum and elytra brighter. Body length 7.3–9.6 mm	***S.congruus*** (Morawitz, 1862)

## Supplementary Material

XML Treatment for Synuchus congruus

## ﻿Additional information

### Use of AI

No use of AI was reported.

### Author ORCIDs

Mikalai Kazulka https://orcid.org/0009-0000-3888-839X

Alexander Anichtchenko https://orcid.org/0000-0001-8512-830X

Oleg Aleksandrowicz https://orcid.org/0000-0001-8176-0674

### Data availability

All of the data that support the findings of this study are available in the main text.
